# Internet-based interdisciplinary therapeutic group (Grupo Interdisciplinar Online, GIO) for perinatal anxiety and depression—a randomized pilot study during COVID-19

**DOI:** 10.1007/s00737-023-01412-2

**Published:** 2023-12-27

**Authors:** M. Gomà, E. Arias-Pujol, E. Prims, J. Ferrer, S. Lara, V. Glover, M. Martinez, A. Llairó, N. Nanzer

**Affiliations:** 1 Faculty of Psychology, Education and Sports Sciences Blanquerna, Ramon Llull University (URL), Barcelona, Spain; 2 Department of Perinatal Care, Bruc Salut Clinical Psychology Center, Barcelona, Spain; 3 Roquetes-Canteres Primary Care Center, Catalan Public Health, Barcelona, Spain; 4 Institute of Reproductive and Developmental Biology, Imperial College London, London, UK; 5 Child and Adolescent Psychiatry Service, Geneva University Hospitals, Geneva, Switzerland

**Keywords:** Depressive-anxiety symptomatology, Perinatal, Interdisciplinary, Online therapeutic group, Brief psychodynamic intervention, COVID-19

## Abstract

Early interventions may promote reductions in mothers’ anxiety-depression (AD) symptoms and improvements in their offspring. This longitudinal randomized research was conducted to assess the effects of interdisciplinary online therapeutic groups (GIO) in at-risk mothers and babies during the COVID-19 pandemic in a disadvantaged neighborhood in Barcelona (Spain). A total of 135 babies were born from March 2020 to June 2021 in a primary healthcare center of Barcelona (Spain). Pregnant woman and new mothers were screened for AD symptomatology through EPDS and STAI questionnaires. Seventy-two of them met high-risk criteria for AD and were included in the study. They were randomly assigned into the two groups of the study: 40 participants were assigned to GIO, the therapeutic group (TG), while 32 of them were assigned to the control group (CG) and received treatment as usual. The course of the mothers’ symptomatology was assessed, as well as the baby’s development at 6 months old in a blind pediatric follow-up. No differences were found in AD between both groups before the intervention. However, we obtained a significant decrease in AD symptomatology (EPDS *p* < .001; STAI state *p* = .015 and STAI trait *p* < .001at 6 months of life) after the intervention in the TG compared to the CG. Pediatric follow-up at 6 months demonstrated significant differences between groups in babies’ development assessment (manipulation *p* = .003; language *p* < .001; sociability *p* < .001). The GIO helped to ensure healthy development of the baby and reduction of the mothers’ depressive-anxiety symptomatology.

## Introduction

Prevalence of perinatal depression and anxiety is high, around 10–20% (Pawluski et al. 2017), and has a direct impact on pregnancy and the fetus, with an increased risk of negative repercussions in neurodevelopmental, cognitive, and emotional problems (Gentile 2017; Glover 2014; Lautarescu et al. 2020; Osborne et al. 2018). Neuroscience highlights the changes in the brain in the transition to motherhood (Barba-Müller et al. 2019), and these have been associated with peripartum mental illness with detrimental effects on the health of the mother, the child, and the family (Pawluski et al. 2021). Mothers with depressive symptomatology may be less available for the interaction with the baby, which can cause difficulties in cognitive and emotional development. The absence of maternal depression was reported as a protective factor in the development of psychotic disorders (Keskinen et al. 2018).

Loneliness and isolation can affect depressive/anxiety (AD) symptomatology in pregnancy, especially in disadvantaged neighborhoods, where the rate of perinatal depressive/anxiety symptomatology is twice as high (Nanzer 2009), recently confirmed in the Roquetes neighborhood in Barcelona which had a pre-COVID-19 rate of 48% on these categories of symptomatology (Gomà et al. 2020b).

Early interventions looked very promising to improve outcomes in mothers and babies (Sleed et al. 2023). Mothers diagnosed with perinatal anxiety and depression could potentially benefit from them to decrease stress and anxiety symptoms and promote their ability to read infant cues appropriately (Hakanen et al. 2019). The need for preventive interventions has also been highlighted by the Geneva team (Nanzer et al. 2012a, b), who designed specific psychotherapies for pregnant women and mother-infant dyads (Moayedoddin et al. 2013) applying intervention measures broadly used (Van Niel and Payne 2020; Hessami et al 2022). Psychodynamic group therapies during the perinatal period have been less explored, especially in an interdisciplinary, Internet-, and community-based approach (Thapa et al. 2020). In perinatal intervention research, little has been mentioned on the effect of child development (Sleed et al. 2023). A recent meta-analysis (Adina et al. 2022) mentioned that it is important to find interventions that can simultaneously prevent and treat maternal postnatal depression as well as have an effect on the mother–child bond and child development. During the pandemic lockdown, it was essential to explore new therapies, such as telemedicine and group approach (Buultjens et al. 2021). Thapa et al. (2020) considered it vital to use interdisciplinary approaches and Internet-based therapeutic interventions in the context of the COVID-19 crisis and to focus on prevention and treatment in both mothers and babies.

This study aimed to assess a specific care for mothers-to-be and mother-baby dyads, in a new interdisciplinary online therapeutic group intervention (Grupo Interdisciplinar Online, GIO). The hypothesis was that the GIO would reduce depressive-anxiety symptomatology in the mothers, improving their global functioning and the development of their babies.

## Material and methods

### Design and assessment

**Fig. 1:**
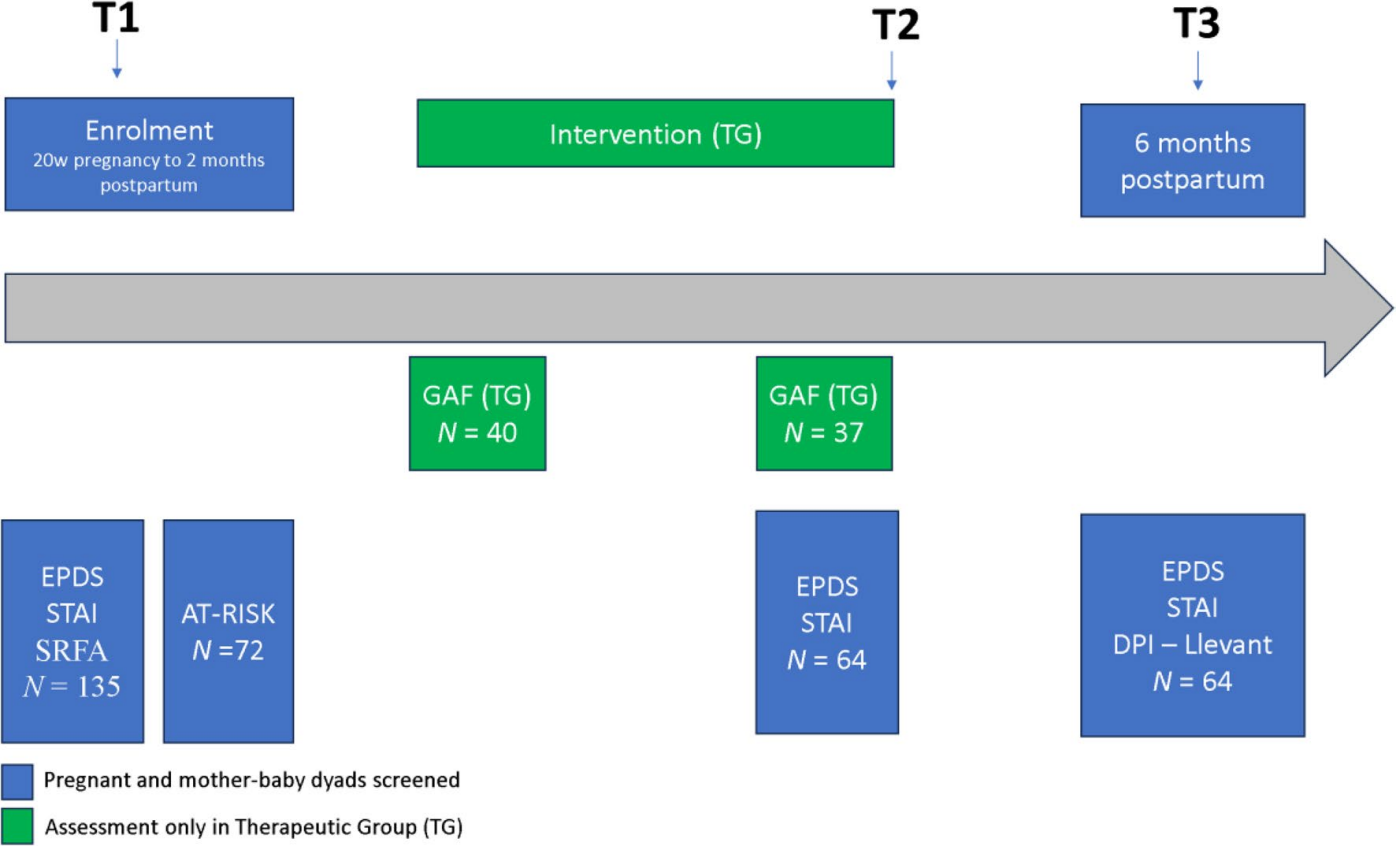
Assessment from 20 weeks pregnancy to 6 months postpartum in the three test moments described: T1, T2, T3

A longitudinal randomized study was conducted with pregnant and new mothers, screened with depressive and anxious symptoms. The TG received GIO intervention, while the CG received treatment as usual and was composed of women in a waiting-list. The main outcomes were collected until 6 months postpartum. Figure 1 shows the design and the flowchart of the study.

#### Recruitment

The study took part in a primary care center (PCC) (Roquetes-Canteres, Barcelona, Spain), in a disadvantaged neighborhood: high unemployment, low income, gender inequalities, high care burden, small apartments, lack of support, high risk of mental disorder, high COVID-19 rates (Health Quality and Assessment Agency of Catalonia (AQuAS) Covid indexes during lockdown 2020; Barcelona Public Health Agency (ASPB) 2019) , 48% of pregnant women with depressive-anxiety symptomatology, and many risk factors (Gomà et al 2020a, b).

A total of 135 babies were born in this center between March 2020 and June 2021. All pregnant women were consulting PCC and sexual and reproductive healthcare professionals (SRHCPs), and all mothers and newborns that met the inclusion criteria during this period on their first pediatric visit were invited to participate. Signed informed consent was obtained from all participants by the midwife or the nurse who met the pregnant women or new mothers at the center. After explaining the study aims, socio-demographic questionnaires (Ad-hoc) for maternity, EPDS, and STAI questionnaires were administered through an interview with the midwife or pediatric nurse.

##### Inclusion criteria

All women with EPDS ≥ 9 results and/ or STAI-trait and state > 39 with two types of inclusion criteria were used: (1) pregnant women from 20 to 32 weeks gestation and (2) mothers with newborn babies aged between 0 and 2 months.

In the TG, data were included for participants who attended a minimum of five sessions.

##### Exclusion criteria

(1) Not fluent in Spanish; 2) major psychiatric disorder in mothers or pregnant women 3) severe abnormalities in the newborn 4) mothers younger than 18 were also excluded 5) women receiving other therapies. These women and families were referred to specialized public services.

#### Allocation and assessment

When results indicated risk (EPDS ≥ 9 and/or STAI (either state or trait) > 39), participants were randomly allocated to either the TG or CG. Allocation was done by an independent pediatric nurse, based on the date of the child’s birth. In the TG, two independent psychologists carried out observation of the first and last group sessions to determine GAF ratings (DSM-IV) (American Psychiatric Association (APA) 2002) .

Measures of EPDS and STAI were repeated after the last intervention session (TG), with a brief satisfaction questionnaire (open questions) by an independent psychologist. The CG was also evaluated by a pediatric nurse when the baby was 4 months old.

A follow-up blinded assessment was carried out by pediatric professionals to TG and CG when the baby was 6 months old: mother’s EPDS and STAI questionnaires and a babies’ development scale.

### Instruments

The following instruments were used to evaluate depressive and anxiety symptoms:

#### EPDS

The Edinburgh Postnatal Depression Scale (Cox et al. 1996) is the most reliable screening tool to assess perinatal depression (Van Niel and Payne 2020). It was initially validated to assess depressive symptomatology in the postnatal period. It was later validated as a reliable means of identifying symptoms of depression in pregnant women (Nanzer 2009). A score equal to or greater than 9 is considered an indication of risk. A version previously validated in a Spanish population was used (Ascaso et al. 2003) .

#### STAI Questionnaire

The State-Trait Anxiety Inventory (Spielberger et al. 1970) is one of the instruments used most frequently to measure anxiety. It differentiates between situations causing temporary anxiety, and permanent anxiety as a trait. It has been shown to be a valid, sensitive instrument for the measurement of anxiety in different populations and has been validated in a Spanish adult population (Guillen-Riquelme and Buela-Casal 2014) . A score equal to or greater than 39 is commonly used as a cut-off point. It was previously used to screen mothers in the perinatal period in several studies (Hessami et al. 2022), specially by the Geneva School (Moayedoddin et al. 2013).

#### The Global Assessment Functioning (GAF) for the TG (Axis V of the DSM-IV-TR)

Pre- and post-intervention group. Scores range from 0 to 100 to measure patients’ current degrees of impairment in psychosocial, education, or occupational functioning. It forms part of the multiaxial system for psychiatric diagnosis (American Psychiatric Association (APA) 2002).

#### Sociodemographic and risk factors data questionnaire (SRFDQ)

This is an ad hoc questionnaire created by our expert professional team expanding on risk factors published in scientific literature. This instrument was previously used in research on risk factors in the same disadvantaged neighborhood (Gomà et al. 2020b).

#### Developmental Pediatric Instrument—DPI Llevant (Catalan Public Health– Institut Català de la Salut ICS) (Catalan Public Health, 2008)

Blinded evaluation of baby development, measuring four domains to detect difficulties. Each domain covers specific development stages expected each month from 0 to 2 years old in manipulation, language skills, sociability, and physical development. It was validated in a population of Catalan infants aged 0–2 years and is widely used in pediatric public health.

### Characteristics of participants

**Fig. 2:**
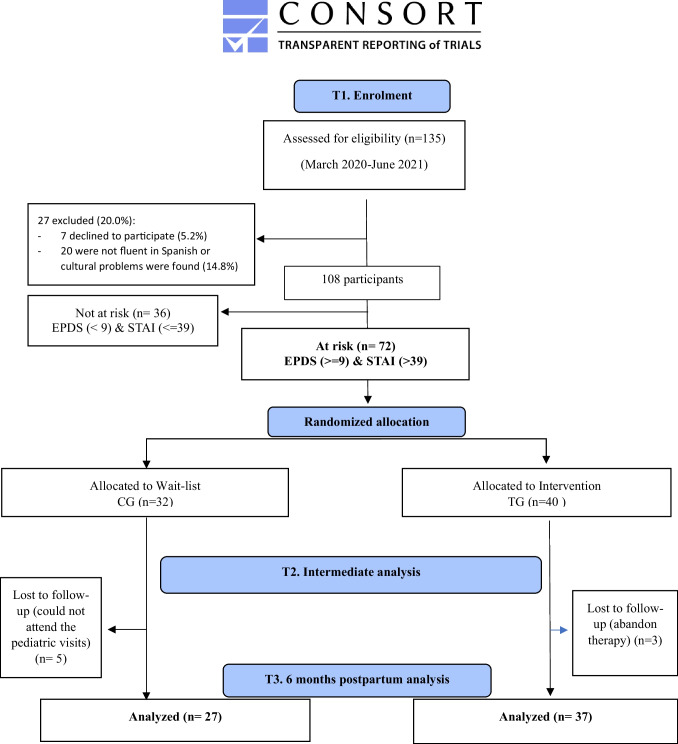
Flow of participants to the study

A total of 135 pregnant/mother-baby dyads were screened in PCC Roquetes-Canteres (Barcelona, Spain). One hundred eight women signed the informed consent. From them, 72 women were detected at risk with depressive-anxiety symptomatology (66.7%). Figure 2 illustrates the flow of participants. TG was composed by 40 mothers (pregnant and/or mothers with 0–2-month-old babies) who were treated with GIO in this period. The women had an average age of 31.27 years (standard deviation 6.42; min 19; max 42 years) and 54.1% of participants were immigrants. Approximately 15% of participants had language (not fluent enough in Spanish to understand correctly or problems to express themselves) or cultural problems (fearing to be influenced by a different culture in raising their child or were not able to participate due to the lack of the husbands’ permission to relate with others). Individual assistance, such as translators, were offered by the PCC during the visits.

The CG contained 32 mothers (pregnant or with newborns aged between 0 and 2 months old) with depressive-anxiety symptomatology, who followed treatment as usual. An average age of 32.35 years (standard deviation 6.25) was found and 62.96% were immigrants.

### Description of the GIO intervention

GIO was offered to mothers who experienced depressive-anxiety symptomatology in their PCC visits following the inclusion criteria. Patient groups are composed of five to eight pregnant women (prenatal intervention) or mother-infant dyads (postpartum intervention), led by two health professionals from different specialties: psychotherapist and midwife or pediatric nurse. After an initial individual telephone session with the group psychotherapist, eight weekly online group sessions lasting 90 min are scheduled, using a Zoom assistant screen. These are closed groups, it means that once the GIO were initiated, they were closed to new participants (Gomà et al. 2020a).

Each session starts by attending to physical issues during 10 min each session (prevention on pelvic floor pain, babies’ development exercises, postural hygiene, baby carrying positions, etc.), relaxation (breathing techniques, visualization and contact with one’s own emotions), and singing (lullabies from their own experience) and moved on to the expression of emotions and feelings between the participants and therapists. A short presentation of about 20 min is given on mothers’ themes and worries gathered from a previous session. The final part was 60 min of free discussion, where women can express their distress, insecurities, and fears. Quality of the intervention is guaranteed by the small size of the group but also because of video attendance, guidance of the exercises, and participation during the sessions.

The central issue combines contributions from the pediatric nurse or midwife with psychological/emotional transgenerational elements in parenthood (Nanzer et al. 2012b), such as lullabies from the memories of maternal grandmothers and infant massage.

The different specialties are combined with an interdisciplinary perspective to provide containment to mothers or future mothers in a holistic bio-psycho-social response to their needs. Working in an interdisciplinary manner grants the opportunity to offer comprehensive care of the body and mind for each pregnant woman and postpartum mothers as an emotional and physical cocoon that could contain her suffering and that of her baby (Coromines 1998; Corominas et al. 2008; Lopez-Corvo 2020; Nanzer et al. 2012a, b). Every group session ends with a 30-min post-group meeting solely with professionals to discuss the emotions experienced and to organize the following session.

In the pregnant mothers’ group, the baby accompanied the sessions from the womb. The mother communicates with the fetus continuously and shares her emotions with the baby and the group. The prepartum GIO intervention concludes before the birth. With the postpartum GIO intervention, the baby was also present, frequently in the mother’s lap where they could hear, see, and participate.

### Data analysis

The statistical package SPSS Statistics for Windows V20 (IBM 2011) was used to carry out a repeated measures ANOVA comparing the TG and CG at three scheduled time points in the study of depressive-anxiety symptomatology. Comparison of means was conducted for independent samples between the CG and the TG to assess inter-group differences in relation to depressive-anxiety symptomatology and development at 6 months, applying *U* Mann–Whitney. A comparison of means for related samples was performed to study the course of symptomatology in each of the groups studied, along with changes in the mothers’ global functioning in the TG (GAF). A chi-square test was applied to compare TG and CG on the different domains of baby development at 6 months old. We used an alpha level of 0.05 for all statistical tests.

## Results

### Sociodemographic description and associated risk factors: TG vs CG

With a confidence level of 95% and a margin of error of 5%, the sample size was representative of the studied population for sociodemographic characteristics. For most risk factors, there were no significant differences between groups. However, there were differences in the level of education (*p* = 0.005; less education in CG) and a history of mental health (MH) problems (*p* = 0.013; more MH problems in TG), as shown in Table 1.

**Table 1:** Baseline sociodemographic description and associated risk factors in TG vs CG

	TG		CG		
	Mean	SD	Mean	SD	Mean comparison (*p* value)
Age (mother)	31.3	6.4	32.2	6.1	0.567
Accumulated risk factors	3.6	2.6	4.5	2.5	0.145
	N	%	N	%	Chi-square (*p* value)
Living in one room	7	18.9	5	18.5	0.968
Migration	16	43.2	12	44.4	0.924
No basic education	4	10.8	11	40.7	0.005*
Unemployment	18	48.6	17	63	0.256
Previous miscarriage	8	21.6	8	29.6	0.465
Voluntary pregnancy termination	3	8.1	7	25.9	0.053
Lack of partner support	5	13.5	7	25.9	0.209
Lack of family support	8	21.6	10	37	0.176
Lack of social support	4	10.8	6	22.2	0.214
History of MH problems	18	48.6	5	18.5	0.013*
Psychotropic drugs	1	2.7	4	14.8	0.075
Previous experience of violence	6	16.2	2	7.4	0.293
Mistreatment in infancy	2	5.4	4	14.8	0.202
Stressful events in the last 24 months	25	67.6	21	77.8	0.370

*p < *0.05*

#### Participation in therapy

An average of 7.16 sessions of participation in the groups was found (SD 0.89; mode 7, minimum 5 sessions and maximum 8).

### Depression, anxiety, and global function of mothers

No significant differences were found in the TG when comparing pregnant and postpartum interventions in the mothers’ depressive-anxiety symptomatology baseline, applying *U* Mann–Whitney test, as shown in Table 2.

**Table 2:** Means (standard deviation) of the AD symptomatology in pre and postpartum participants

	EPDS			STAI state			STAI trait		
	CG	TG	*p* value	CG	TG	*p* value	CG	TG	*p* value
Pregnancy									
T1	11.6 (3.5)	10.7 (2.5)	0.548	76.3 (13.2)	86.5 (10.1)	0.067	66.1 (19.2)	68.9 (19.6)	0.581
T2	12.2 (3.5)	5.3 (2.9)	> 0.001*	77.4 (15.8)	65.2 (17.4)	0.067	66.4 (20.9)	59.2 (13.3)	0.236
T3	12.8 (5.1)	6.1 (4.0)	0.001*	81.3 (14.7)	74.9 (22.8)	0.648	71.1 (18.7)	52.9 (16.4)	0.005*
Postpartum									
T1	11.1 (4.5)	13.4 (4.1)	0.540	77.0 (14.5)	84.0 (16.2)	0.081	64.5 (17.3)	69.9 (23.1)	0.256
T2	12.5 (4.6)	6.3 (4.2)	0.001*	79.2 (13.4)	84.9 (15.4)	0.087	69.2 (17.8)	63.7 (18.6)	0.460
T3	12.9 (4.8)	5.2 (3.6)	0.001*	83.0 (12.1)	68.2 (24.8)	0.160	71.1 (25.3)	51.5 (24.7)	0.023*

*p < 0.05

**Fig. 3:**
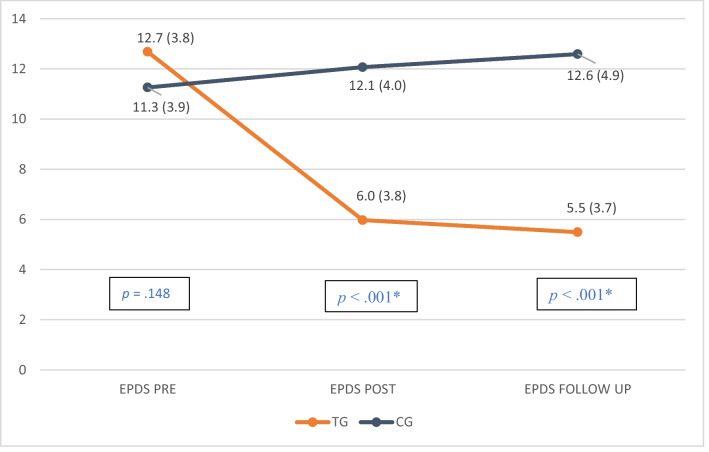
Means of depression scores (standard deviation) over the course of the intervention and follow-up (6 months-old)*.* TG, therapeutic group; CG, control group; *p* < 0.05

No differences were found before intervention when comparing both TG and CG in depression and anxiety trait symptomatology (EPDS *p* = 0.148; STAIT T *p* = 0.493). However, there were marked, statistically significant differences between the TG and CG in EPDS scores when applying a lineal general model of repeated measures at T1 (pre-treatment *p* = 0.148), T2 (post-treatment *p* < 0.001), and at the follow-up T3 (*p* < 0.001) when the baby was 6 months old, with a Mauchly’s W *p* = 0.120 (see Fig. 3).

**Fig. 4:**
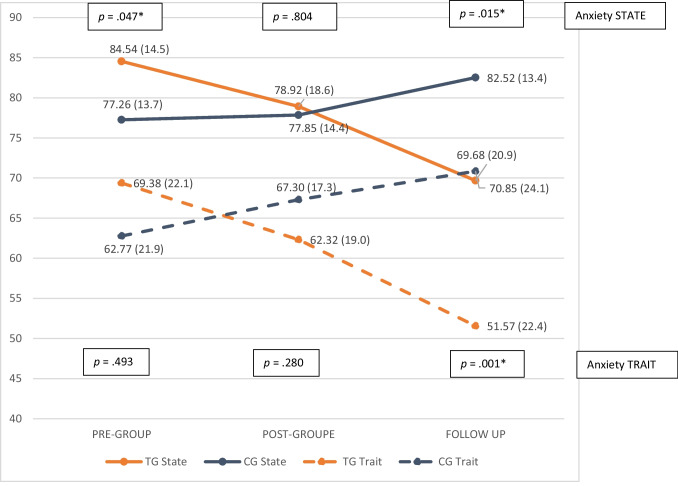
Means of anxiety scores (state and trait) over the course of the intervention and follow-up. TG, therapeutic group; CG, control group. *p < 0.05

Anxiety symptoms followed a similar trend when applying a repeated measures ANOVA, with a significant reduction in the TG when the baby was 6 months old in both STAI state (multivariate contrast *p* = 0.017; Mauchly’s W *p* < 0.001) and trait (multivariate contrast *p* = 0.002), due to a Mauchly’s W ( *p* < 0.001) (see Fig. 4).

In the TG, using a comparison of means for related groups, we obtained statistically significant differences (*p* < 0.001) with an improvement of 18.3 (SD 4.9) points in the mean of mothers’ global functioning (GAF from DSM-IV).

### Baby development

We obtained no significant differences in pre- and postpartum intervention in the number of baby difficulties at 6 months postpartum when applying a Mann–Whitney *U* test (*p* 0.359).

In terms of baby development, significant differences between groups were found (Table 3). In the TG, only one baby showed an alteration in physical development (2.7%), while in the CG, eight children were assessed to have some alteration (33.3%). Three dyads from the TG relocated, and it was not possible to follow on their development.

**Table 3:** Developmental difficulties observed in the pediatric evaluation at 6 months

	TG (*N* = 37)		CG (*N* = 24)		Chi-square
	N	%	N	%	p
Physical development	7	18.9	9	37.5	0.107
Manipulation	1	2.7	7	29.2	0.003*
Language	0	0	7	29.2	0.000*
Sociability	0	0	9	62.5	0.000*

*p < 0.05

### Baby development and mother symptomatology

Pearson correlations were applied to observe the relationship between STAI and EPDS scores and number of difficulties in the babies’ development. In the whole sample (*N* = 64), the higher the EPDS score at 6 months old, the greater the number of difficulties in the babies’ development (Pearson correlation 0.318; *p* = 0.013). This correlation was not found when studying the TG alone (Pearson 0.036; *p* = 0.832). No significant correlations were found in the anxiety scores.

When applying a Mann–Whitney *U* test, a similar course was found between treatment in antenatal and postpartum (pregnant-women TG and postpartum-dyad TG), in EPDS, STAI, and baby difficulties.

At the end of the therapeutic group sessions, the babies had relaxed, taken pleasure in the relationship, and improved sleep and rest patterns, along with feeding recommendations. Changes were noted in the pediatric follow-up; mother hypervigilance of the babies decreased, and better attendance to appointments and increased biopsychosocial well-being in the mother-baby relationship were observed.

Following the group sessions, which allowed the mothers to understand their babies’ needs, they reported that the babies cried less and were more easily consoled. The mothers explained that they were acquiring the ability to read infant cues appropriately.

All the pregnancies in the TG went to term with good resolution despite the warning regarding probable prematurity given to each of the pregnant women by the health professionals. The intrauterine growth of all the babies was adequate, even in the case of one baby who presented an initial developmental delay (3rd percentile).

Due to the nature of the intervention, no harm was done to participants or their babies. Results in both mother and baby health outcomes and patient satisfaction were very positive.

## Study limitations

This study had some limitations that must be addressed: the sample of patients was small. TG and CG had similar baseline characteristics except for level of education (lower in CG) and history of mental health problems (higher in TG), but the overall accumulation of risk factors in both groups were similar. Further studies are needed to expand on the data to corroborate the tendencies observed and to increase understanding and effectiveness of online interdisciplinary therapeutic groups in disadvantaged neighborhoods. Future research with larger samples and a longer follow-up could explore the durability of the benefits of the intervention.

## Discussion and conclusion

There are few studies on child development outcomes together with mother–child bonding in parenting interventions related to perinatal depression on mothers (Adina et al. 2022). Our study provides positive results on the development of babies, the symptomatology of mothers, and the bond established between them through a group intervention about parenting. This is specially significant in the population studied due to its characterized high vulnerability and their known epigenetic impact (Glover 2023).

As hypothesized, interdisciplinary online therapeutic group (GIO) reduced depressive and anxiety symptomatology in the mothers, improved their global functioning, and resulted in significant improvements in the development of the baby. Mothers improved family and social relationships, self-care, and care of the baby, while strengthening ties with the health center and building trust with the professionals in the network.

Scientific literature highlights the negative repercussions of prenatal maternal depression-anxiety on the baby’s neurodevelopment (Gentile 2017; Glover 2014; Keskinen et al. 2018; Lautarescu et al. 2020; Osborne et al. 2018; Srinivasan et al. 2020). In this pilot study, in the blind pediatric follow-up when the baby was 6 months old, a third of the children in the CG presented difficulties in one or more developmental areas, with statistically significant differences between groups. These results show an indicator of the challenge that public health has to face, in this case, the high vulnerability of the low-income population. The very low dropout rate in our online therapeutic group indicates good acceptance and the feasibility of the intervention, which is not common in the perinatal field (Iturralde et al. 2021). In the satisfaction questionnaire, the mothers explained how they felt supported, empowered, and understood. They perceived professional empathy and tailored care, key aspects identified in perinatal period women’s experiences of care (Westgate et al. 2023). As many authors have pointed out, lockdown increases isolation and reduces access to care (Bao et al. 2020; Giesbrecht et al. 2021; Ozamiz-Etxebarria and Idoiaga Mondragon 2020; Xiang et al. 2020). GIO intervention, possibly due to being online and having an interdisciplinary perspective (Thapa et al. 2020), has reduced the effects of isolation and facilitated access to professional care.

There is a clear perception of the need for preventive interventions focused on parenthood and promoted access to the mothers’ internal world, as reported by the Geneva team (Nanzer et al. 2012a,b; Moayedoddin et al. 2013). The pattern of evolution of depressive-anxious symptomatology is surprisingly similar in pregnancy intervention and postpartum intervention. We hypothesize that although they are two very different vital moments, the basis of group psychological work focused on parenthood is similar, as well as the methodology (interdisciplinary, online, number of sessions, organization of sessions, associated task, etc.). These results are similar to the meta-analysis performed by Adina et al. (2022) in which positive effects are found in both antenatal and postnatal parenting interventions.

The inclusion of new interdisciplinary working methods was effective in terms of prevention in mothers with depressive-anxiety symptomatology and their babies from both the physical and psycho-emotional points of view. GIO is a perinatal specific intervention, with personalized care and professional empathy.

The high cost-effectiveness of perinatal preventive interventions and their strong socio-economic impact are well known (Heckman 2017). These findings may help policy-makers and mental health professionals to take advantage of the benefits of telemedicine (Cantor et al. 2022) and interdisciplinary approaches in their work.

## Data Availability

The datasets generated and/or analyzed during the current study are available in the Open Zenodo repository: Zenodo. 10.5281/zenodo.7369696.

## Abbreviations

*CT*: Control group
*EPDS*: Edinburg Postpartum Depression Scale
*GAF*: Global Assessment Functioning
*GIO*: Grupo Interdisciplinar Online
*ICS*: Catalan Public Health–Institut Català de la Salut
*MH*: Mental health
*PCC*: Primary care center
*SRFDQ*: Sociodemographic and risk factors data questionnaire
*SRHCP*: Sexual and reproductive healthcare professionals
*STAI*: State-Trait Anxiety Inventory Questionnaire
*TG*: Therapeutic group
