# Correction to: Internet-based interdisciplinary therapeutic group (Grupo Interdisciplinar Online, GIO) for perinatal anxiety and depression—a randomized pilot study during COVID-19

**DOI:** 10.1007/s00737-024-01418-4

**Published:** 2024-01-11

**Authors:** M. Gomà, E. Arias-Pujol, E. Prims, J. Ferrer, S. Lara, V. Glover, M. Martinez, A. Llairó, N. Nanzer

**Affiliations:** 1 Faculty of Psychology, Education and Sports Sciences Blanquerna, Ramon Llull University (URL), Barcelona, Spain; 2 Department of Perinatal Care, Bruc Salut Clinical Psychology Center, Barcelona, Spain; 3 Roquetes-Canteres Primary Care Center, Catalan Public Health, Barcelona, Spain; 4 Institute of Reproductive and Developmental Biology, Imperial College London, London, UK; 5 Child and Adolescent Psychiatry Service, Geneva University Hospitals, Geneva, Switzerland

**Correction to: Archives of Women’s Mental Health**

10.1007/s00737-023-01412-2

The corresponding author of the published article entitled “Internet based interdisciplinary therapeutic group (Grupo Interdisciplinar Online, GIO) for perinatal anxiety and depression—a randomized pilot study during COVID-19” is requesting the 2nd affiliation to be the 1st affiliation because the University of the second affiliation is covering the open access fees.

The original article has been corrected.

